# Annatto Oil Loaded Nanostructured Lipid Carriers: A Potential New Treatment for Cutaneous Leishmaniasis

**DOI:** 10.3390/pharmaceutics13111912

**Published:** 2021-11-11

**Authors:** Marianna Araújo Ferreira, Renato Ferreira de Almeida Júnior, Thiago Souza Onofre, Bruna Renata Casadei, Kleber Juvenal Silva Farias, Patricia Severino, Camilo Flamarion de Oliveira Franco, Fernanda Nervo Raffin, Túlio Flávio Accioly de Lima e Moura, Raquel de Melo Barbosa

**Affiliations:** 1 Laboratory of Drug Development, Department of Pharmacy, Federal University of Rio Grande do Norte, Natal 59012-570, Brazil; marianna_af@hotmail.com (M.A.F.); fernanda.raffin@ufrn.br (F.N.R.); 2 Department of Clinical and Toxicological Analysis, Federal University of Rio Grande do Norte, Natal 59012-570, Brazil; renato.junior.104@ufrn.edu.br; 3 Biochemistry and Molecular Biology Department, Federal University of Viçosa (UFV), Viçosa 36570-900, Brazil; tsonofre@unifesp.br; 4 Institute of Physics, University of São Paulo, USP, São Paulo 05508-090, Brazil; casadei@if.usp.br; 5 Academic Unit of Biology and Chemistry, Center of Education and Health, Cuité 58175-000, Brazil; kleber.farias@ufcg.edu.br; 6 Institute of Technology and Research (ITP), Aracaju 49010-390, Brazil; patricia_severino@itp.org.br; 7 Phytotechnical Department, Brazilian Agricultural Research Corporation (EMBRAPA), Brasilia 70770-901, Brazil; camilo-urucum@hotmail.com

**Keywords:** annatto oil, *Bixa orellana* L., cutaneous leishmaniasis, nanostructured lipid carriers (NLC), lipid nanoparticles

## Abstract

Annatto (*Bixa orellana* L.) is extensively used as food pigment worldwide. Recently, several studies have found it to have healing and antioxidant properties, as well as effective action against leishmaniasis. Therefore, the purpose of this study was to incorporate the oil obtained from annatto seeds into a nanostructured lipid carrier (NLC) and evaluate its physicochemical properties and biological activity against *Leishmania major*. Nanoparticles were prepared by the fusion-emulsification and ultrasonication method, with the components Synperonic™ PE (PL) as the surfactant, cetyl palmitate (CP) or myristyl myristate (MM) as solid lipids, annatto oil (AO) (2% and 4%, *w*/*w*) as liquid lipid and active ingredient, and ultra-pure water. Physicochemical and biological characterizations were carried out to describe the NLCs, including particle size, polydispersity index (PDI), and zeta potential (ZP) by dynamic light scattering (DLS), encapsulation efficiency (EE%), thermal behavior, X-ray diffraction (XRD), transmission electron microscopy (TEM), Electron Paramagnetic Resonance (EPR), cytotoxicity on BALB/c 3T3 fibroblasts and immortalized human keratinocyte cells, and anti-leishmaniasis activity in vitro. Nanoparticles presented an average diameter of ~200 nm (confirmed by TEM results), a PDI of less than 0.30, ZP between −12.6 and −31.2 mV, and more than 50% of AO encapsulated in NLCs. Thermal analyses demonstrated that the systems were stable at high temperatures with a decrease in crystalline structure due to the presence of AOs (confirmed by XRD). In vitro, the anti-leishmania test displayed good activity in encapsulating AO against *L. major*. The results indicate that the oily fraction of *Bixa orellana* L. in NLC systems should be evaluated as a potential therapeutic agent against leishmaniasis.

## 1. Introduction

Presently, more than one billion people in the world have been affected by Neglected Tropical Diseases (NTDs), which is considered to be an important public health issue [1,2]. Leishmaniasis is an emerging uncontrolled NTD [3]. There are twelve million people who are currently infected worldwide, culminating in a high mortality rate (26,000 to 65,000 deaths/year). Epidemiological research indicates that there are 350 million people at risk from a zoonotic infection disease caused by Leishmania parasites in 102 countries, areas, or territories, and large parts of the world, including the European continent [2,4]. In Spain, for example, leishmaniasis is endemic and of increasing incidence [5].

There are different kinds of Leishmania infection: Visceral Leishmaniasis (VL) and American Cutaneous Leishmaniasis (ACL), which is subdivided into Cutaneous Leishmaniasis (CL), Diffuse Cutaneous Leishmaniasis (DCL), and Mucosal Leishmaniasis (ML) [6]. Although not lethal, ACL is the most common, affecting about one million people per year, and can cause severe deformities [7].

The latest report from the World Health Assembly on leishmaniasis highlighted an urgent need for its control and monitoring in endemic areas, and increased investment in research and development for new drugs with better bioavailability and lower costs. In addition, the same report stressed the need for an existing diagnostic protocols regimen, especially for the worst case of Leishmania, VL [8].

Leishmania parasites are transmitted to humans through the bite of female sandflies of the genera Lutzomyia and Phlebotomus. Like many protozoan parasites, Leishmania parasites have a digenetic lifecycle: an extracellular mobile stage (promastigote), exclusive to the invertebrate host, and an intracellular (amastigote) non-mobile stage in a mammalian vertebrate host [9], where the parasites infect cells of the phagocytic mononuclear system, where the macrophages are the main cellular compartment in the vertebrate host [10].

Currently, the first choices for antileishmanial drugs are Meglumine Antimoniate (Glucantime^®^) and Sodium Stibogluconate (Pentostam^®^); an alternative treatment is administered using Amphotericin B, Miltefosine, Paromomycin, and Pentamidine [11,12,13]. Although these drugs have been used for many years, numerous problems have been reported, such as a high volume of adverse effects, with toxicity as the essential factor, low adherence to treatment when parenteral administration is required, an extended period of use, the high cost of some treatments, resistance (developed by the parasite), and a lack of the medication itself. Considering the problems related to the drug therapy currently employed in the treatment of leishmaniasis, the search for new assets capable of overcoming such limitations and promoting a cure for patients has become increasingly necessary [3,14,15,16]. The use of secondary metabolites from nature to develop new drugs is a promising area that deserves further research and development, but as yet, has not been fully explored [17].

Annatto (*Bixa orellana* L.) is a native plant of the tropical regions of America [18]. Annatto seeds are typically composed of 50% carbohydrate, 12–17% protein, 9–13% water, 5–7% ash, 2–5% lipid, and 1–6% various pigments [19]. Also present are alkaloids, flavonoids, other carotenoids, gallic acid, orelline, di, mono and sesquiterpenes, and palmitic and linoleic acids. Bixin represents about 80% of the compounds present in the annatto oil fraction (AO). Besides these compounds, the oil of annatto seeds is rich in tocotrienol-type antioxidants (~90% delta-tocotrienol, 10% gamma-tocotrienol) [20]. The FDA has approved annatto and it is frequently used in foods and beverages, cosmetics, pharmaceutical products, and in natural dyes in the textile industry [21].

The development of new nanotechnology platforms can help to overcome the limitations of standard drugs against Cutaneous Leishmaniasis by concentrating on the reduction of administered doses, targeting drugs, increasing drug stability, and overcoming problems with immunological recognition delivery [22,23]. Furthermore, nanocarriers can improve therapeutic efficacy by delivering leishmanicidal drugs in macrophage-rich organs such as the bone marrow, liver, and spleen. Macrophages, the main phagocytic cells involved in leishmaniasis infections, can internalize nanoparticles in the size range of 50–500 nm [24].

Lipid nanoparticles (LNP) comprise solid lipid nanoparticles (SLN), nanostructured lipid carriers (NLCs), and lipid drug conjugates (LDCs). SLNs are carriers composed of solid lipids at room temperature, while NLCs are obtained from both solid and liquid lipids. As Leishmaniasis disseminates through the lymphatic and vascular systems, infecting monocytes and macrophages in the liver, spleen, bone marrow, and lymph nodes, NLCs appear to be an interesting strategy for leishmanicidal treatment, due to their tendency to target the lymphatic system [24]. NLCs have interesting properties and are considered “NanoSafe”, mainly due to their excellent stability, biocompatibility, superior loading efficiency for hydrophobic drugs (natural [25,26,27] or synthetic [28,29]), and low nanotoxicity [30,31]. The versatile nature of their inner core, high encapsulation efficiency, and the low amount of solid content makes them attractive candidates.

Thus, this study aimed to incorporate AO into an NLC suitable for the treatment of CL. This study evaluated physicochemical parameters pertinent to the formulation and stability of the obtained nanoparticles. In addition, the in vitro cytotoxicity assays and leishmanicidal activity of AO in NLCs were evaluated as a potential source for the development of phytopharmaceutical products.

## 2. Materials and Methods

### 2.1. Materials

Crodamol (cetyl palmitate, CP) and Synperonic™ PE (PL) were received as free samples from Croda (Campinas, SP, Brazil). Myristyl myristate (MM) was donated by Dhaymers Fine Chemicals (Taboão da Serra, SP, Brazil). Chr. Hansen (São Paulo, SP, Brazil) supplied the oily fraction from *Bixa orellana* L. seeds. Hansen (São Paulo, SP, Brazil). Mouse embryo BALB/c 3T3 fibroblasts and immortalized human keratinocyte cells (HaCaT) were purchased from Rio de Janeiro Cell Bank (Rio de Janeiro, RJ, Brazil). All other chemicals and solvents were of analytical grade. Deionized water (18.2 MΩ cm) was obtained from a Waters ultrapure water system.

### 2.2. Methods

#### 2.2.1. Preparation of Nanoparticles

**Figure 1:**
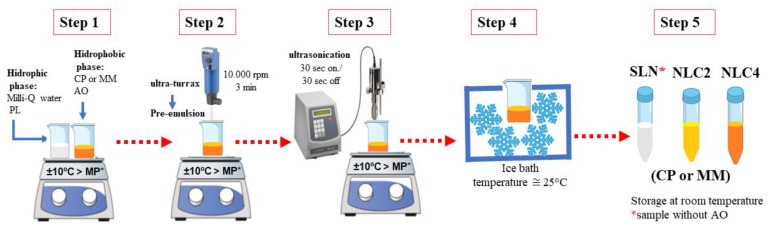
Schematic representation of the method used to prepare lipids nanoparticles (SLN and NLC).

Nanoparticles were prepared by the hot emulsion technique and ultrasonication with composition, provided in Table 1 [32,33]. The preparation of lipid nanoparticles involved five steps as presented in Figure 1: (1) Heating: CP and MM were heated at 10 °C above their melting point, and for NLC, AO was solubilized into the lipid previously melted (CM or MM). The PL aqueous solution was also heated at the same temperature as the oil phase. (2) Pre-emulsion production: the pre-emulsion (O/W) was obtained using a Turrax mixer (IKA Werke GmbH & Co. KG; Staufen im Breisgau, Germany) under high speed (10,000 rpm) for 3 min. (3) Homogenization process: Pre-emulsion was homogenized by tip ultrasonication at 20 kHz for 15 min (15 s on and 15 s off) in a Vibra-Cell ultrasonic processor (Sonics and Materials; Newtown, CT, USA). (4) Cooling: All samples were cooled in an ice bath. (5) Packaging: Samples were placed in falcon tubes at room temperature. The presence of AO favours the production of NLCs disorganized internal structure due to the mix of different liquid lipid at room temperature [34].

#### 2.2.2. Particle Size, Polydispersity Index, and Zeta Potential

Particle size, polydispersity index (PDI), and zeta potential (ZP) were all measured by photon correlation spectroscopy (PCS), where samples were diluted (1:100) in Milli-Q water at 25 °C with a scattering angle of 173° by ZetaSizer Nano ZS (Malvern^®^ Instruments, Malvern, UK).

#### 2.2.3. Transmission Electron Microscopy (TEM)

TEM was applied to investigate the morphological aspect of the nanoparticles. For this purpose, samples were analyzed using a JEOL JEM-2010HC TEM (JEOL Co. Ltd.; Tokyo, Japan). Samples were diluted (1:50) in distilled water and placed on a 200 Mesh Cu grid (Electron Microscopy Sciences; Hatfield, PA, USA). A uranyl acetate aqueous solution (1%, pH 4.0) was mixed with the nanoparticles to improve contrast and then air-dried at room temperature. The images were obtained for freshly prepared samples (one week of storage).

#### 2.2.4. Thermal Analysis

The thermal profile of the samples was evaluated through thermogravimetry. For this, weight loss versus temperature was investigated using DTG-60 (Shimadzu^®^; Tamboré, Barueri, SP, Brazil). The samples were previously frozen at −20 °C for six hours and then freeze-dried for 24 h (Martin Christ Freeze-dryers; Osterode am Harz, Germany). The freeze-dried samples were analyzed under temperatures ranging from 25–500 °C, under a nitrogen atmosphere, a gas flow rate of 50 mL/min, and a heating rate of 10 °C/min, using alumina crucibles. The samples also were analyzed by Differential Scanning Calorimetry (DSC) using STA 449 F3 Jupiter^®^ (NETZSCH Thermal Analysis; São Paulo, SP, Brazil) under an atmosphere of nitrogen, 50 mL/min flow, under a temperature range of 25 °C to 200 °C, and a heating rate of 10 °C/min. Indium was used to calibrate the equipment (temperature and enthalpy).

#### 2.2.5. X-ray Powder Diffraction (XRD)

XRD analysis was performed to confirm the crystalline arrangement, and to study the polymorphism of the lipids and nanoparticles. The diffractograms were obtained using an X-ray diffractometer (D2 Phaser, Bruker Corporation; Madison, WI, USA), where CuKα (*λ* = 1.54 Å) radiation was used as X-ray source. The interlayer spacings were calculated from the reflections using the Bragg’s equation (Equation (1)),

(1) $$d=\frac{\lambda }{2\;sen\;\theta }$$

where *λ* is the wavelength of the incident X-ray beam and θ is the scattering angle. The parameter *d*, also termed interlayer spacing, is the separation between a particular set of planes of the crystal lattice structure [35].

For the analysis, the freeze-dried samples were placed in the aluminum sample supports and analyzed from 5° to 45°, with an acquisition time of 0.1 s, at 30 kV of operating voltage, and a current of 10 mA, using a Lynxeye detector.

#### 2.2.6. Electron Paramagnetic Resonance Spectroscopy (EPR)

EPR is a spectroscopic technique that operates in the microwave region (range 9 to 10 GHz). It consists of detecting, indirectly, one or more unpaired electrons (transitions of electronic spin states) of chemical compounds (paramagnetic molecules), which, when subjected to a magnetic field, reorient their electronic spins according to their magnetic spin moments [36,37]. The EPR of spin labels incorporated into lipid vesicles or amphiphilic aggregates has been broadly used to analyze the viscosity and polarity of the microenvironment where they are monitored [38]. EPR (Bruker EMX spectrometer (Bruker BioSpin GmbH, Billerica, MA, USA) was employed to identify the effect of AO on the mobility and organization of the lipid core of the nanoparticles. The stearic acid-derivative spin is labelled at the 5th and the 16th carbon of the acyl chain (5- SASL and 16- SASL). The stearic acid labelled at the 5th carbon atom monitors the nanoparticle region closer to its water interface. In contrast, the 16 C-atom labels provide information about the nanoparticle core, close to the end of the lipid (CP or MM) hydrocarbon chains.

5- SASL and 16- SASL were inserted into the lipid milieu of SLNcp, SLNmm, NLCcp4, and NLCmm4, to monitor the molecular arrangements of CP or MM (with and without AO) at 37 °C, up to 0.8 mol % (relative to total lipid concentration). The spectra were analyzed in terms of an empirical parameter, ∆H_0_ (width of the central line), at a temperature between 15 and 50 °C [39]. The contribution of both order and mobility of the spin-label inserted into lipid phases can be displayed using this parameter. Lower ∆H_0_ values correspond to either lower order, or higher mobility, or both. We use the term organization to refer to the sum of both contributions [38].

#### 2.2.7. Encapsulation Efficiency (EE%)

EE% of AO was determined by the indirect ultrafiltration method. Samples were initially diluted in ultrapure water (1: 500), passed through 10 KDa pore filtration units, and then centrifuged (7000 rpm, 20 min, 10 °C). The supernatant containing the encapsulated oil was diluted (1:3) in ethanol to allow the particles to rupture and quantified by spectrophotometer at 452 nm using Varian Cary^®^ 50 UV–Vis (Palo Alto, Santa Clara, CA, USA) [40]. Calculations were performed using the following Equation (2):

(2) $$\mathrm{EE}\;\left(\%\right)=\frac{\mathrm{Encapsulated}\;\mathrm{AO}}{\mathrm{Total}\;\mathrm{AO}\;\mathrm{added}}\times 100\;$$

#### 2.2.8. In Vitro Cytotoxicity Assay

The cytotoxicity of free and encapsulated AO against BALB/c 3T3 and HaCaT cells was investigated by MTT assay. Cells were cultured in a 75 cm² flask in DMEM, supplemented with 10% (*v*/*v*) fetal bovine serum or bovine calf serum and antibiotics (streptomycin (10 mg/mL), penicillin (10,000 U/mL) and amphotericin B (25 µg/mL)), under a humidified atmosphere at 37 °C with 5% CO_2_ (Revco™, Thermo Fisher Scientific; Waltham, MA, USA). Cells were inoculated onto a 96-well plate at 1.9 × 10^4^ cells per well for BALB/c 3T3 and 2.9 × 10^5^ cells per well for HaCaT and were incubated under humidity at 37 °C with 5% CO_2_ for 24 h. The medium was then replaced with nanoparticles (SLNcp, NLCcp2, and NLCcp4) solubilized in a fresh medium at different concentrations (25–300 µg/mL). The plate was incubated in the same conditions as previously described for 24 h. MTT was added and after four hours of incubation, DMSO was added to the wells to solubilize formazan crystals. Cell viability was quantified at 540 nm using an Epoch Microplate Spectrophotometer (Biotek, Winooski, VT, USA) [32,41].

#### 2.2.9. In Vitro Antileishmanial Assay

An antileishmanial assay was carried out as reported by Bastos et al. (2017) but with some modifications. Macrophages of the RAW 264.7 strain were kept in a humid oven at 37 °C with 5% CO_2_, with RPMI medium, pH 7.2, supplemented with sodium bicarbonate (2 mg/mL), HEPES (25 mM–pH 7.2) L-glutamine (2 mM), penicillin (0.02 mg/mL), gentamicin (20 μg/mL), and 10% (*v*/*v*) fetal bovine serum [42,43].

*L. major* cells were grown in Grace’s medium (pH 6.5) supplemented with L-glutamine (2 mM), penicillin (0.02 mg/mL), and 10% (*v*/*v*) inactivated fetal bovine serum and sterilized by 0.2 μm pore nitrocellulose membrane filtration, then kept in a dry stove at 25 °C and maintained every three to four days. The macrophages were initially transferred (200 µL) to a 96-well plate at 1 × 10^5^ macrophages/well and incubated at 37 °C for 24 h. The *L. major* cells were centrifuged at 1500× *g* for ten mins at 4 °C and then inoculated (200 µL) onto the plate at a concentration of 15 × 10^5^ *L. major*/well. The plate was again incubated at 37 °C for 24 h.

At the end of the incubation, each well was washed three times with RPMI base for the removal of Leishmania cells that did not infect the macrophages. RPMI (200 µL) was added, and the plate was incubated at 37 °C for 24 h. The metabolized medium was removed, and a fresh medium was added to each well. DMSO (0.1%) was then added to the control wells, macrophages in culture medium and infected macrophages without treatment were maintained in internal control wells, and for the test wells, Amphotericin B (0.3 and 3.125 µg/mL), Glucantime^®^ (200 and 400 µg/mL), unencapsulated AO, and SLNcp, NLCcp2, and NLCcp4 (2 and 5 µg/mL) were added.

The plate was incubated at 37 °C for 48 h. After incubation, each well was washed three times with RPMI base to remove the substances. Posteriorly, RPMI (50 µL) plus 0.05% of SDS was added to the wells, and the plate was exposed to room temperature for thirty minutes to promote the lysis of macrophages. Grace’s supplemented medium was then added to disrupt lysis and the plate was incubated at 25 °C for up to seven days. Finally, resazurin (20 μL–1 mM) was added and the plate was again incubated for one hour, followed by colorimetric analyses (Spectramax^®^ M5, Molecular Devices; San Jose, CA, USA), performed at wavelengths of 570 nm and 600 nm. The results were established considering the control wells with untreated infected macrophages as maximum viability, and by comparing the others in percentage terms.

#### 2.2.10. Statistical Analysis

Statistical analyses were performed using a student’s *t*-test, ANOVA, and a Tukey-Kramer post-test performed on GraphPad Prism 6.0 software (GraphPad Software 2012; San Diego, CA, USA). The level of significance (α) was 5%.

## 3. Results

### 3.1. Particle Size, Polydispersity Index, and Zeta Potential

**Figure 2:**
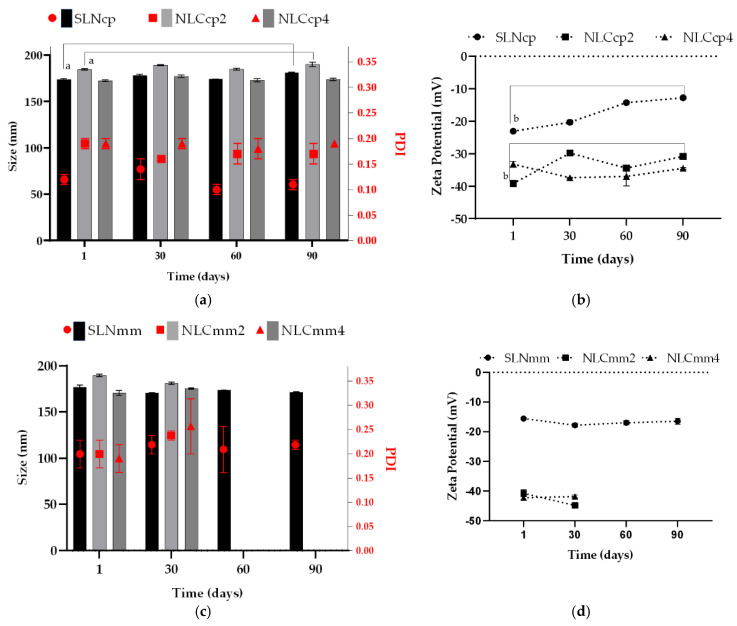
Particle size, polydispersity index, and zeta potential of CP (**a**,**b**) and MM (**c**,**d**) nanoparticles in different storage times. Statistics: Mean ± SD, *n* = 3. The results of each sample obtained throughout the storage were analyzed through ANOVA, with a Tukey-Kramer post-test. ‘**a**’ represents a statistical difference of day one vs. day ninety of the SLN and NLCcp2 for particle size; ‘**b**’ day one vs. day ninety of SLN and NLCcp2 for zeta potential. Significance was considered *p* < 0.05 for all tests. Observed a phase separation of NLCmm2 and NLCmm4 at day 60 and 90 (size, PDI, and zeta potential data are presented in Supplementary Materials, Table S1).

The nanocarriers were analyzed for particle size, PDI, and ZP at 1, 30, 60, and 90 days (see Figure 2). The ultrasonication method promotes the reduction of the oily droplet size to the sub-micron scale, and typically forms particles of size less than 500 nm [44].

PDI is a parameter that gives information about the quality of the system’s dispersion, distinguishing between mono and polydisperse systems. The low PDI values (ranging from 0.10 ± 0.01, and 0.27 ± 0.06) indicate a monodispersed system with a unimodal distribution, which corroborates with DSC and XRD analyses, as discussed below, which demonstrates a uniform dispersion of AO in the lipid matrix. However, SLNcp and NLCcp2 significantly altered their sizes between 1 and 90 days (ANOVA, *p* < 0.05), suggesting a tendency to increase particle diameters as the ZP values decreased. In addition, Figure 2 displays the stability of the SLNmm over the studied period without a significant (*p* > 0.5) change in size, PDI, or ZP in contrast to NLCmm (2 and 4) that, after 30 days, presented visual phase separation. Figure 2 also displays similar zeta potential values for samples with, and without, AO for both structural lipids (CP and MM), but SLNs (i.e., without AO) presented low zeta potential values (in modulus), indicating that the presence of AO is an important factor related to changes in zeta potential values. Over the storage time, the ZP of NLCcp2 and NLCcp4 varied by around −30 mV, while the SLNcp formulation underwent a significant (*p* < 0.05) reduction (in modulus) throughout storage, indicating a reduction in the stability of the system. However, the sample NLCcp4 (with a higher concentration of AO) presented smaller size variation throughout storage, indicating that not only the presence, but the quantity of this oil can be a determinant factor in the maintenance of nanoparticle size.

The pH of the colloidal dispersion (between 5.0–6.0 for all samples) can also contribute to zeta potential alteration due to the composition of AO. The main components are apocarotenoids as bixin (pKa = 4.9), norbixin (pKa = 4.7), and fatty acids as palmitic (pKa = 9.7), stearic (pKa = 10.15), oleic (pKa = 9.85), and linoleic (pKa = 9.24) [45,46,47]. In general, carotenoid radical cations (Car*^+^) can be formed by electron transfer from the carotenoid to Lewis acidic sites on a surface. Car*^+^ is a weak acid that can lose an H^+^ to form a proton, less neutral radicals, resulting in the carboxyl group (COO^−^), explaining the system’s most negative charge. Furthermore, fatty acids containing 18 carbons (C18) have an ion-dipole strong interaction between the carboxylic groups when the values of environment pH are similar to the compound’s pKa. On the other hand, an additional observation is the high tendency of compound aggregation when the pH is lower than pKa with the possible formation of crystals or precipitates [47]. A zeta potential above ±30 mV is a strong indication of a system’s stability because it reflects high repulsion of the particles, preventing their aggregation or sedimentation [48].

### 3.2. Morphology

**Figure 3:**
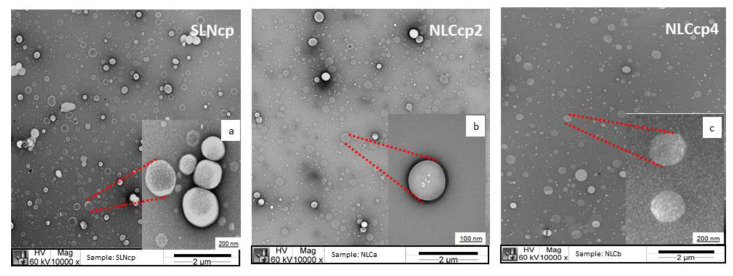
Transmission electron microscopy of SLNcp (**a**), NLCcp2 (**b**), and NLCcp4 (**c**) display the morphology and size of the lipid-based nanoparticles. Images with 10,000× and 60,000× (red highlight) of magnification give polydispersity and details of some nanoparticles, respectively.

Figure 3a–c displays micrographs of the nanoparticles based on cetyl palmitate as a solid lipid, SLNcp, NLCcp2, and NLCcp4. According to the images, it was possible to see well-defined structures that were approximately spherical. The sizes of the nanoparticles were similar for the three samples analyzed and were around 200 nm, corroborating with the data obtained by the DLS technique. Similar results were obtained for SLNmm, NLCmm2, and NLCmm4 (Figure S2 in Supplementary Materials).

### 3.3. Thermal Profile

**Figure 4:**
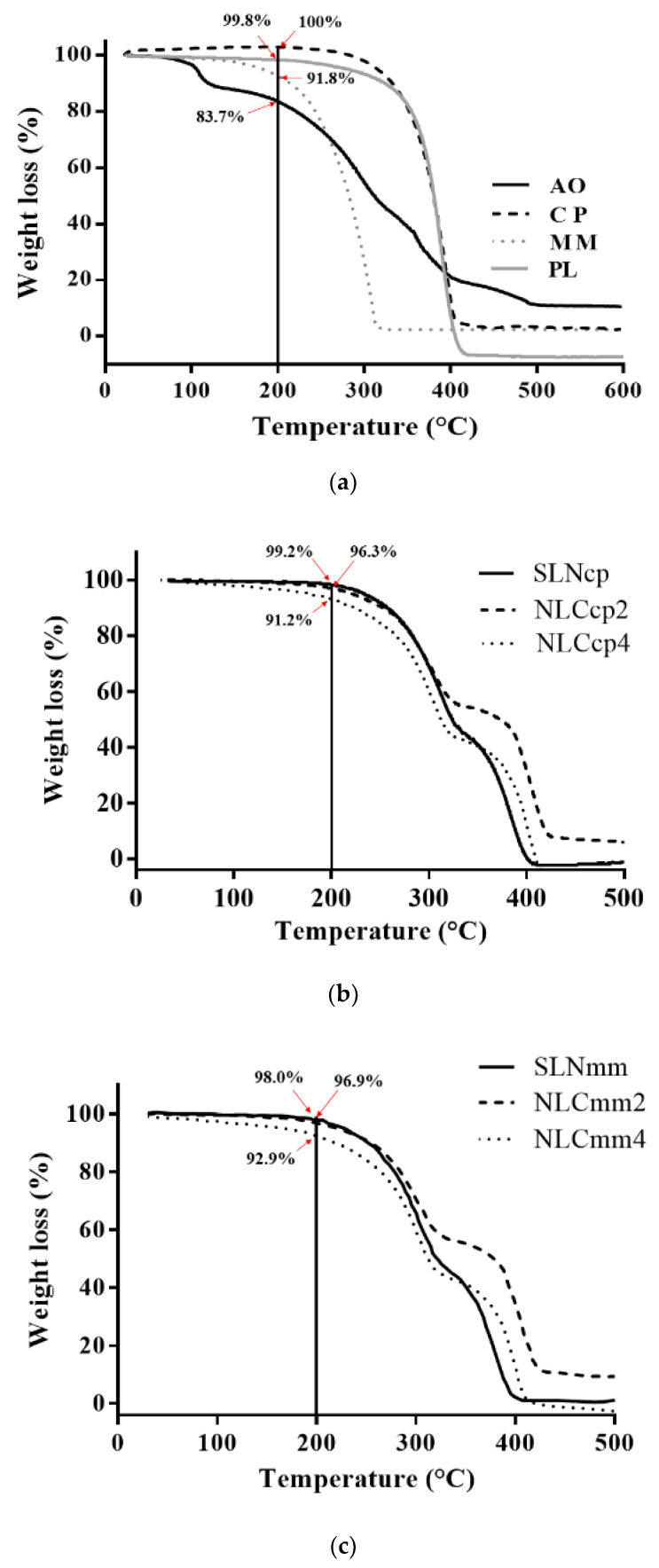
Thermogravimetry analyses of pristine compounds (**a**), cetyl palmitate-based nanoparticles (**b**), and myristyl myristate based nanoparticles (**c**).

Thermal characterization is essential for physicochemical evaluation of lipid-based nanoparticles. The results can provide information about stability through to thermal behavior, crystallinity, and polymorphism of nanoparticle ingredients [49,50]. Figure 3 and Figure 4 report the comparison between the thermogravimetric and calorimetric measurements performed on raw materials and nanoparticles.

Figure 4a displays a loss of mass in a single event starting at 191 °C, 120 °C, and 245 °C for CP, MM, and PL, respectively. Nevertheless, the AO presented mass loss in four steps. The first occurred between 32 °C to 101 °C with an 11.5% mass loss due to the evaporation of volatile components. Then, a loss of 42.8% occurred between 103 °C to 242 °C, this being related to the material that started to degrade. The third reduction occurred between 247 °C and 323 °C, where 17.9% of the oil mass was lost due to the degradation of organic compounds. Then, the final loss of 18.7% was observed between 324 °C and 479 °C, with the carbonization of the material (Figure 4a); these results are in agreement with the results obtained by Bitencourt and co-authors in 2018 [51].

The four thermal events of the AO do not appear in Figure 3b,c. This behavior can be explained by the molecular dispersion of the oil within the NLCs promoting a less ordered structure, as demonstrated by DSC analysis. In addition, the encapsulation of AO in lipid carriers promoted greater thermal stability compared to free AO.

**Figure 5:**
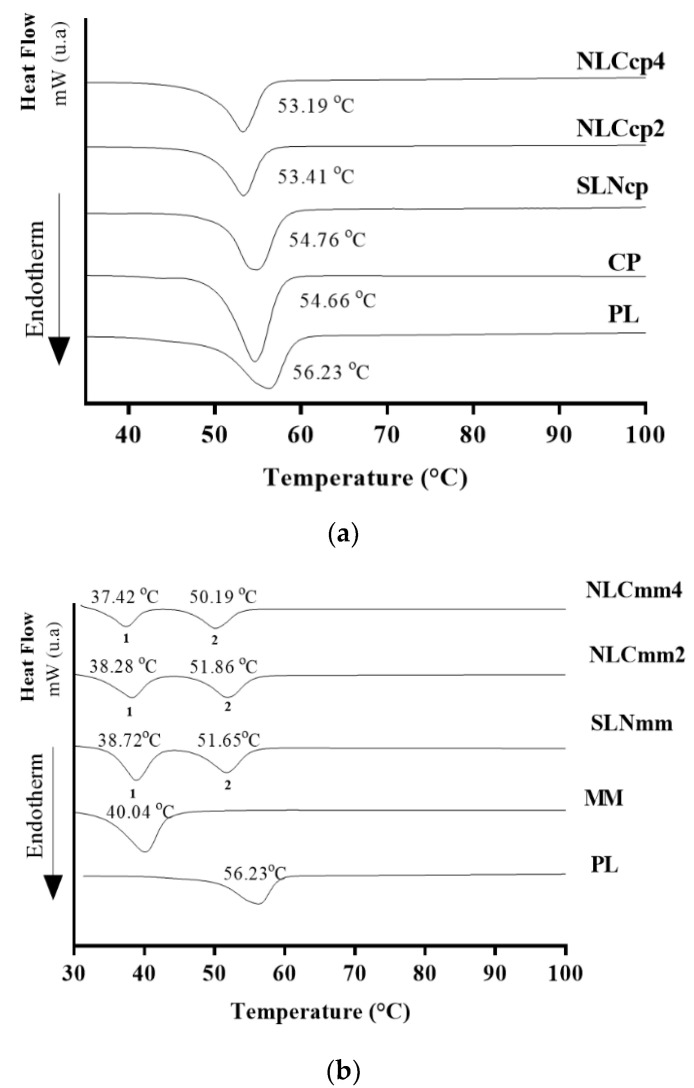
DSC curves of lipid nanoparticles and their compounds. Lipid nanoparticles produced with cetyl palmitate (**a**) and myristyl myristate (**b**). The melting points of the first and the second peak of samples were represented by numbers 1 and 2 respectively.

DSC curves regarding raw materials (CP, MM, PL, and AO) and lipid nanoparticles (SLN and NLC) are presented in Figure 5, and they corroborate with the literature [32,49,52]. All nanocarriers presented melting points below the temperatures of the raw materials (around 57 °C) and above 37 °C. The range of endothermic events, and the respective enthalpy observed for the lipid nanoparticles and their components are presented in Figure 5 and Table 2. However, it can be observed that AO is one of the main factors responsible for the structural modifications in the nanostructured lipid carriers as corroborated by encapsulation efficiency (EE%), XRD, and EPR analyses.

Changes in the thermal profile with the displacement of peaks and the shift of melting point for all NLCs with reduction on the melting enthalpy were observed, being the highest reduction observed for samples with 4% of AO, if compared with structural lipids (CP or MM respectively) or unloaded SLN (Figure 5a,b). This suggests a homogenous distribution of AO in the lipid matrix [49]. As a result, there is an increase in the defects of the lattice [53]. Besides, these shifts of the peaks to lower temperatures are related to the increase in the amounts of AO. Moreover, the absence of new thermal events suggests a change in the structural organization of the NLCs due to the amorphization or molecular dispersion of the oil into the lipid matrix [54] without the formation of a new chemical entity.

The decrease in the melting point of the second peak of the NLCmm can also be explained by the fact that the MM melts before and contributes to the solubilization of part of the system, which does not occur with CP. NLCs have several advantages over SLNs, one of which is the disorderly crystalline arrangement caused by liquid lipid, which allows for the more efficient retention of the active compound, prolonging its release time/rate [55].

Techniques such as DSC, XRD, and EPR can help to elucidate the arrangements of crystalline structures, and the degree of order of the lipids present in SLN and NLC. The composition and production parameters of nanoparticles must be evaluated regarding the formation of different crystalline forms that in general provide significant physical and chemical changes regarding the shape, solubility, melting point, and crystallization of these formed structures. Modifications in the fractions of polymorphs present in the composition of nanoparticles can lead to a reduction, displacement, or change in the melting temperature of the system [33].

Table 2 illustrates endothermic parameters calculated from DSC curves obtained to lipid nanoparticles and their structural lipids. Melting point peak shifts were observed that can relate to information about the modifications of the lipid polymorphic state (from crystalline β to metastable form β’) during the cooling of the melted lipid. Also, enthalpy reduction was observed in lipid nanoparticles compared to pristine lipids, due to the formation of a new structure indicating their decreased crystallinity [49,56].

### 3.4. Structural Characterization of Nanoparticles

#### 3.4.1. X-ray Powder Diffraction (XRD)

The presence of polymorph crystals in SLN/NLC’s structures provides significant physical and chemical changes in form, solubility, and melting point, affecting properties related to spreadability, encapsulation capacity, product degradation, and consequently, the release profile of the obtained structures [57,58].

The production of SLN/NLC involves the recrystallization of particles by cooling the samples. Depending on the speed of cooling of the nanoparticles, the appearance of polymorphic forms α, β’, and β occurs. In general, long chain lipids undergo crystallization with two or three different phases detected (α to β’ or α, β’ to β) [59].

The polymorphic form α is considered the most unstable form. It occurs due to rapid cooling, providing polymorphic hexagonal crystals. The β forms (parallel triclinic polymorphs) appear in slower cooling processes with the lipid rearrangement being more ordered and stable, causing a low degree of drug encapsulation. The β’ form (perpendicular orthorhombic structure) is considered an intermediate form between α and β polymorphic forms [57].

**Figure 6:**
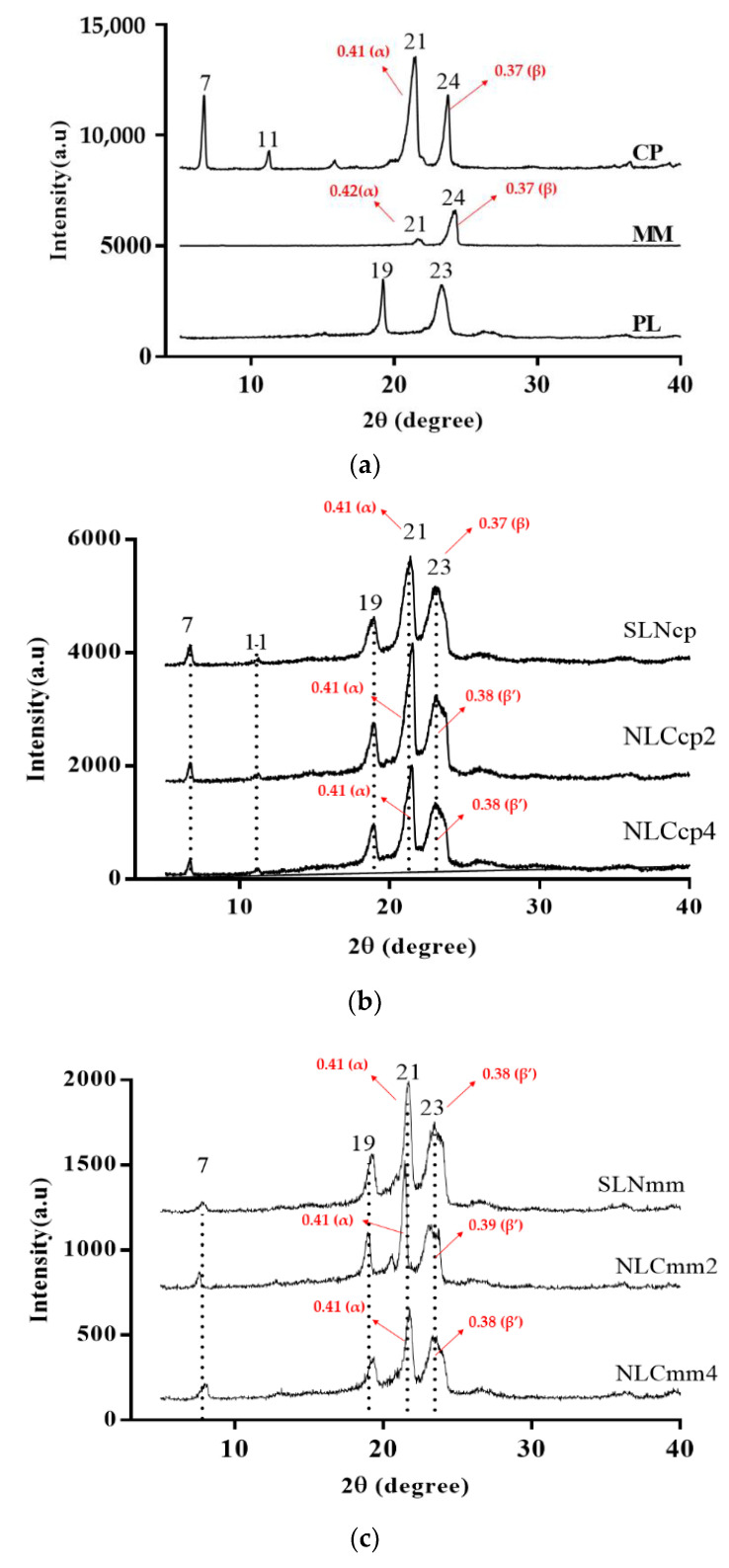
Diffractograms of raw material (**a**) used to produce cetyl palmitate (**b**) and myristyl myristate (**c**) nanoparticles. Note: α: d = 0.41–0.42 nm, β: d = 0.46 nm, β’: 0.42 < d < 0.43 nm or 0.37 < d < 0.40 nm (main reflections and lattice spacings of the CP, MM and SLN/NLC calculated by Bragg equation data are presented in Supplementary Materials, Table S2).

The lamellae arrangement structures of nanoparticles have been discovered by XRD analysis [35,49,60]. The XRD patterns indicate that for SLN and NLC, the intensity of the peaks was reduced (Figure 6), which means changes in the crystallinity of lipids could have occurred. Amorphization can be observed in Figure 5b,c, mainly due to the structure of nanoparticles and the presence of AO. The slight decrease in the melting enthalpy registered by DSC analysis of AO loaded NLCs are in agreement with the decrease in peak intensity observed by XRD analysis, demonstrating a less ordered structure and lattice defects.

CP and MM display a crystalline structure, as α and β (Figure 6a and Supplementary Materials Figure S3), [50,52,61]. The peak at 19° is characteristic of the surfactant (pristine PL); besides, it also appears in all nanoparticles’ diffractograms and demonstrates the same lamellae arrangement when it is a component of SLN or NLC (Figure 6b,c).

SLNcp and NLCcp exhibited sharp peaks at 2θ scattered angles of 21°, confirming the lipid crystalline nature (Figure 6b). The NLCs promoted slight changes in the peaks, mainly at 23° and 24°, suggesting the modification of the crystal structure due to a less stable matrix with the presence of α and β’forms. Moreover, SLNmm and NLCmm exhibited sharp peaks at 2θ scattered angles of 7°, 19°, 21°, and 23°, also confirming the lipid crystalline nature (Figure 6c).

The small difference between XRD results obtained for MM and CP lipids (Figure 5) can be explained by their hydrocarbon chain length with C14 and C16 (carbon atoms), respectively [53]. The reduction in the intensity of main diffraction peaks was observed and is related to the reduction of the degree of crystallinity of the lipid in its nanoparticulate state, obtaining a more amorphous system. The dispersion of AO in the mixture with the solid lipid became the samples which were more amorphous, indicating a less ordered structure and pronounced lattice defects. This fact is in agreement with thermal analysis results; it is known that minimal change in crystallinity, enthalpy and melting temperature of triglycerides are responsive to display α, β’ and β polymorphic forms, and the slight variation corresponds to the change from the amorphous α-form to the most stable β-form [56].

#### 3.4.2. Electron Paramagnetic Resonance (EPR)

**Figure 7:**
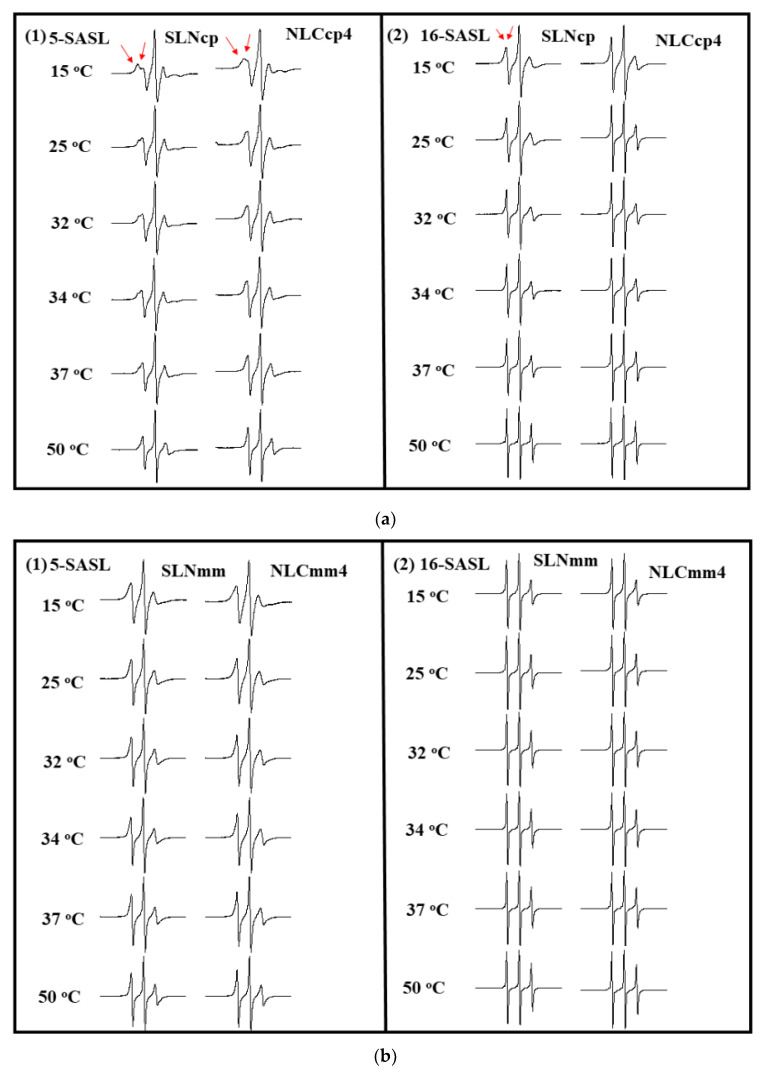
EPR spectra obtained with 5-SASL (**1**) and 16-SASL (**2**), inserted into nanoparticles; SLN and NLC4 (4% of AO) prepared with CP (**a**) and MM (**b**).

Electronic paramagnetic resonance (EPR) is a very powerful biophysical technique for characterizing carrier systems that provides structural and dynamic information of them. In addition, the EPR of spin labels incorporated into lipid vesicles or amphiphilic aggregates has been broadly used to analyze the viscosity and polarity of the microenvironment where they are monitored [33,38,62]. The nitroxide radical (e.g., doxyl-stearic acid, SASL) is the most used spin-label due to its stability over a wide range of temperatures and pH. The unpaired electron of the spin label interacts with the external magnetic field and the nuclear magnetic moment of nitrogen (*I* = 1). This last interaction is called hyperfine interaction and allows for the splitting of energy levels [36,38] responsible for the three characteristic peaks of the nitroxide spectrum, as found in Figure 7. Furthermore, spectral anisotropy is dependent on the orientation of the molecular axis of the nitroxide radical concerning the magnetic field. Thus, it reflects the mobility of this spin marker when incorporated into oriented systems.

**Figure 8:**
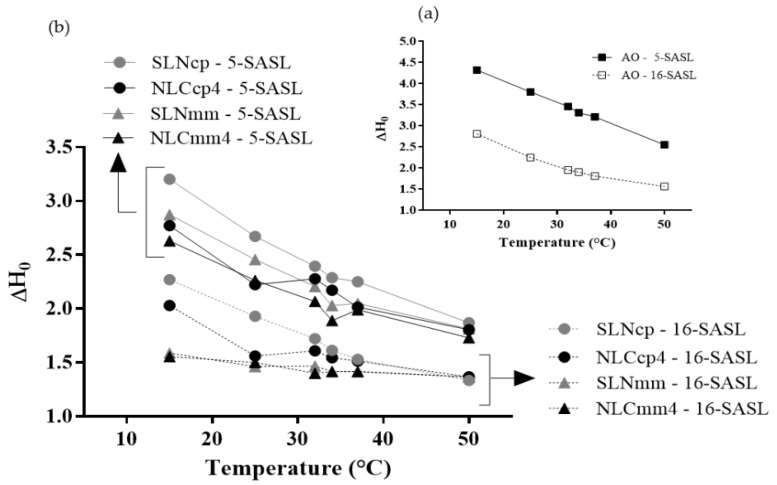
The temperature dependence of the center field linewidth, ΔH_0_, measured on the EPR spectra of 5-SASL and 16-SASL incorporated in AO (**a**) SLN and NLC (**b**) (from 15 to 50 °C).

EPR results obtained with 5-SASL and 16-SASL spin labels after being inserted in bilayers, allowed for the monitoring of different regions of lipid samples. The spectra of both in SLN (cp and mm) and NLCcp4 and NLCmm4 are displayed in Figure 7a,b, while Figure 8a,b depicts the changes in ΔH_0_ as a function of temperature (from 15 to 50 °C).

According to the results presented in Figure 8, all nanoparticles demonstrated a marked fluidity, with a center field linewidth that decreases continuously as one goes deeper into the lipid bilayer. This profile indicates that the alkyl chains are strongly ordered close to the membrane surface (the less fluid region) and strongly disordered in the membrane center (the more fluid region), and this is characteristic of all fluid-phase bilayers. Similar results were achieved by Barbosa and collaborators [32,33]. Comparing CP and MM nanoparticles, those based on CP display an increase in center field linewidth, for two labels. No abrupt transition was observed with temperature.

In the presence of AO, the NLCcp4 was more fluid than SLNcp (Figure 8a,b), as a strong indication of the association of AO with CP for the formation of nanostructured lipid carriers. This effect was even more evident at lower temperatures (35 °C, below phase transition, Figure 8b). Above 35 °C, the center field linewidth of the NLC and SLN were remarkably similar. For MM nanoparticles, these results were not evident, since MM has a melting point lower than CP (Figure 5a), so the labels did not ‘feel’ the difference between the nanoparticles with and without oil. Results concerning the stability of nanoparticles in terms of storage time were discretionary for the choice of samples to continue the work, consequently only samples produced with CP were evaluated for encapsulation percentage, rheological assays, cell viability, and antileishmanial activity.

### 3.5. Encapsulation Efficiency (EE%)

NLCs are characteristically known for their disorganized crystalline structure due to the presence of liquid lipid, allowing the active substance to become trapped in the formed spaces [63]. The quantification of AO was carried out by UV-Vis spectrophotometry at a wavelength of 452 nm (the calibration curve is in Supplementary Materials, Figure S1). Table 3 displays the encapsulation efficiency of the nanoparticles. Despite NLCcp4 demonstrating a lower encapsulation efficiency (EE%), the oil mass encapsulated was 27% more than NLCcp2; this suggests there is an encapsulation limit to the NLCs.

### 3.6. Antileishmanial Activity (In Vitro)

**Figure 9:**
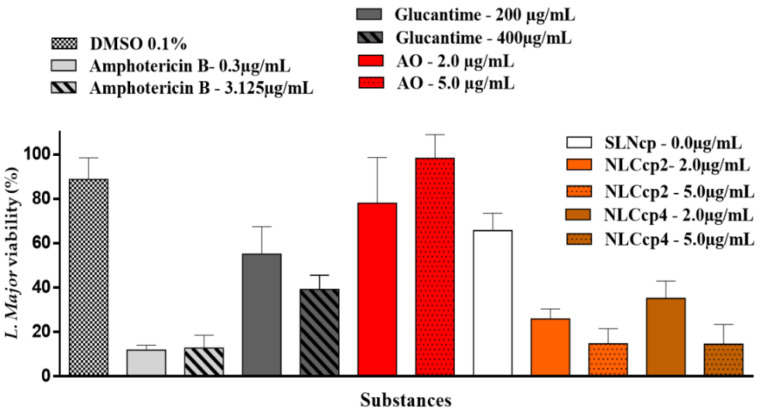
*L. major* viability using macrophages infected with *L. major* and treated with antileishmanial drugs, AO, and nanoparticles with and without AO. Mean ± sd, *n* = 6.

Macrophages infected with *L. major* were treated with Glucantime and Amphotericin B, drugs used in the treatment of leishmaniasis, as well as AO and NLCs (Figure 9). Macrophages were infected with *L. major* and treated with antileishmanial drugs, AO, and nanoparticles with and without AO. After macrophage lysis, the viability of *L. major* was obtained considering the infected macrophages, but not treated as being positive control (i.e., 100% viable).

Free AO was not effective against *L. major*, since almost all cells remained alive after treatment. Previous works demonstrated that the antileishmanial activity (IC_50_) is reached with at least 8.5 µg/mL, almost double that of the tested concentration [64,65]. Furthermore, those experiments were performed with promastigote forms: free cells. Here we tested a lower concentration, based on previous work [66], and we used amastigote forms: an intracellular cell. Therefore, in addition to using a lower concentration, the *L. major* parasite was not directly exposed to treatments. No statistical differences were observed between the concentrations tested (*p* > 0.05). Although the SLN did not have any active compound, it caused an approximate 40% reduction in the parasite cells, presenting a statistically similar result to Glucantime^®^ (200 μg/mL), and superior to that of free AO.

In Figure 9, it can be also observed that there is a reduction of about 35–40% of the parasite cells with the unloaded SLNcp. The antileishmanial activity is not attributed to the lipids but rather to the high surfactant content in the nanoparticles, as discussed below, in the cytotoxicity assay. It was necessary to add 11.7%, *w*/*w* of poloxamer for the entrapment of AO in the lipid matrix of the nanocarriers. Moreover, Yan et al. (2010) [67] discussed the role of poloxamer in drug internalization and as an inhibitor of both P-glycoprotein (P-gp) and cytochrome P450 (CYP3A4), reducing the efflux of drugs from cells. AO-loaded NLCs demonstrated an efficacy of 70–90%, demonstrating that AO has antileishmanial activity and that its incorporation into nanocarriers capable of being internalized by cells is essential for its action against the Leishmania intracellular parasite.

Thus, AO alone does not present activity against Leishmania but was very active in NLCcp formulations (more than two times if compared with the unloaded SLNcp), reinforcing the need for the union of annatto oil and the other of the components of NLCcp to guarantee an effective antileishmanial activity.

NLCcp2 and NLCcp4 at 5.0 μg/mL presented statistical similarity (*p* > 0.05), where both were able to reduce parasitic cells by ≅90%. This data is comparable to the Amphotericin B results. Also, it can be observed that AO encapsulation into NLCcp (2 and 4) is a primary factor in increasing the effectiveness of AO against the parasite.

The absorption process of nanostructures depends on factors such as particle size and surface charge. Jain et al., (2014) presented promising results, where lipid nanoparticles covered with chitosan (~200 nm diameter) were phagocytized and internalized in macrophages previously infected with Leishmania [68].

According to Lopes et al. (2016), the effective antileishmanial activity of lipid nanocarriers goes through an effective internalization process of these nanostructures by macrophages [69]. Therefore, Pires and collaborators (2020) evaluated macrophages’ recognition of their solid lipid nanoparticles by analyzing an uptake assay with fluorescent SLN using thioglycolate-elicited macrophages from male mice [70]. The authors pointed out the lipidic nature, negative charge, and diameter of particles (~100 nm) as the main characteristics that propel the best internalization of the proposed system, and therefore promote a more effective antileishmanial activity.

Amphotericin B is one of the main drugs used in the treatment of cutaneous leishmaniasis but its main downsides are its high toxicity and cost [71]. In contrast, AO is an inexpensive drug; it can be produced by its extraction from annatto seeds. NLCcp2 and NLCcp4 (5.0 μg/mL) demonstrated superior performance compared to Glucantime^®^ (200 and 400 μg/mL) (*p* < 0.05), the first choice antileishmanial drug for the treatment of cutaneous leishmaniasis by the intramuscular (IM) and intravenous (IV) routes, or most recently intralesional administration [72]. However, severe side effects have been reported like nephrotoxicity, hepatotoxicity, acute pancreatitis, cardiac alterations, and reports of varicella-zoster reactivation attributed to glutathione use [73].

### 3.7. Cytotoxicity In Vitro

The skin is a tissue that has one of the greatest diversities of all cell types. Each cell differs according to its metabolism and the way it reacts to stress, generating different responses [74,75]. For this reason, CP lipid nanoparticles (with and without AO) were tested on two of the main cell types found in the skin such as fibroblasts (3T3) and keratinocytes (HaCaT) [76]. Given the serious adverse effects of the mentioned drugs in IM or IV administration with consequent systemic effect, dermal or topical CL treatment may be a promising alternative with local impact.

Regarding the topical application of lipid nanoparticles, Müller and collaborators (2011) described the particles’ occlusive effect on the stratum corneum. The researchers justified that the occlusion occurs due to the development of a lipid film formed by the nanoparticles’ deposition after application on the skin. Adherence is due to hydrophobic interactions occurring between the lipid components of the skin and the formulation, promoting the increase in both the degree of hydration, reinforcement, and repair of the skin, as well as greater penetration of drugs in the deeper layers of the cutaneous tissue [77]. Associated with the ability of NLCs to adhere to the skin, other studies have demonstrated the use of lipid nanocarriers for improving the bioavailability of different drugs against Leishmania [78,79,80,81].

**Figure 10:**
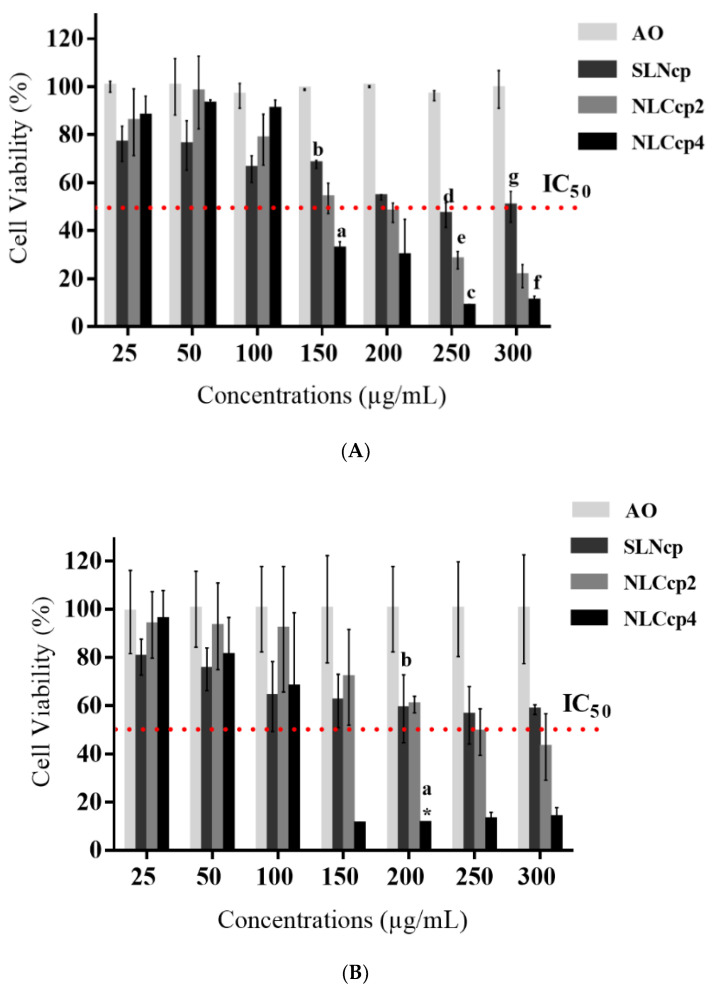
Cytotoxicity of nanoparticles and AO against fibroblasts (**A**) and keratinocytes (**B**). Statistics: (**A**) ANOVA, with a Tukey-Kramer post-test was used in the comparison of the groups of formulations related to each concentration; for that, it was considered: (a) NLCcp4 vs. NLCcp2 (150 μg/mL), (b) NLCcp4 vs. SLN (150 μg/mL), (c) NLCcp4 vs. NLCcp2 (250 μg/mL), (d) NLCcp4 vs. SLN (250 μg/mL), (e) NLCcp2 vs. SLN (250 μg/mL), (f) NLCcp4 vs. SLN (300 μg/mL), and (g) NLCcp2 vs. SLN (300 μg/mL). For the student t test, NLCcp2 vs. NLCcp4 presented no statistical difference between them. Significance was considered *p* < 0.05 for the test. Mean ± sd, *n* = 6. (**B**). ANOVA with a Tukey-Kramer post-test was used in the comparison of the groups of formulations related to each concentration; for that, it was considered: (a) NLCcp4 vs. NLCcp2 (200 μg/mL) and (b) NLCcp4 vs. SLN (200 μg/mL). For the student t test, only NLCcp2 vs. NLCcp4 (200 μg/mL) presented any statistical difference, represented by (*). Significance was considered *p* < 0.05 for the test. Mean ± sd, *n* = 6.

The results from Figure 10A,B displayed no cytotoxic effect caused by AO on fibroblast and keratinocyte cells. For the nanocarriers, only SLNcp’s samples at a concentration ≥ 100 μg/mL promoted a statistically significant reduction of more than 30% of the number of viable cells, being considered cytotoxic to HaCaT and 3T3 cells. On the other hand, NLCcp2 and NLCcp4 samples only at a concentration ≥ 150 μg/mL were cytotoxic to fibroblasts (Figure 10A). Also, Figure 10B displays that NLCs with the highest concentration of AO (NLCcp4) had expressive cytotoxicity in samples with a concentration of ≥150 μg/mL for keratinocytes, unlike samples of NLCcp2 that only displayed a cytotoxic effect at concentration of ≥250 μg/mL.

Besides the type and amount of surfactant, elements such as lipid composition and particle size may also influence any toxicity caused by nanocarriers [82,83]. Barbosa et al. (2013) presented results of the high viability to 3T3 and HaCaT cells by MTT assay when they were submitted to treatment with solid lipid nanoparticles prepared with the MM and CP lipids [32]. However, the formulations contained a poloxamer (PL) concentration ten times lower than the samples demonstrated here. Similar results obtained by Ridolfi 2011 [84] corroborate these findings. Furthermore, it is noteworthy that the sizes, zeta potential, and polydispersity of nanoparticles were very similar in both articles [41]. Currently, more than 200 commercially available cosmetic products contain cetyl esters. In addition, CP is also considered safe for pharmaceutical and food use by the FDA [85]. In addition, Doktorovova et al. (2014) presented a systematic review that demonstrated low cytotoxicity of lipid nanoparticles prepared with CP in different SLNs or NLCs [82].

In the case of PL, it is considered safe and biocompatible [86], so assessing its cytotoxicity alone is not common, even knowing that polymeric surfactants can have their toxicity [87,88]. Recently, it was demonstrated by Utami (2018) [89], an increase in the toxicity of Poloxamer 188 as a function of the increased dose applied to neuroblastoma cell lines. Additionally, studies conducted by Chieng et al. (2009) [90] and Wu et al. (2009) [91] revealed that amphiphilic poloxamers could interact with the lipid bilayer in cell membranes, with or without interrupting its integrity. However, it is not known whether this interaction (poloxamer/lipid bilayer from cells) is beneficial or not, as poloxamers can act as both a membrane sealant and permeabilizer [91]. As membrane sealants, poloxamers have been found to restore the membrane integrity of cells such as fibroblasts [92] and muscle cells [93]. Nevertheless, as a membrane permeabilizer, poloxamer can fluidize the membrane and increase absorption.

Studies have also found that more hydrophobic polymers (with longer PPO blocks) caused more disruption in the lipid bilayer due to more significant interaction with the bilayer lipids present in the cells [90,94]. In addition, results presented by Utami (2018) [89] demonstrated the use of poloxamer-based surfactants at much higher concentrations than CMC provides increased cytotoxicity due to the rapid solubilization of the lipid bilayer caused by the surfactant [90]. Thus, the reduction in cell viability presented for SLNcp samples against fibroblasts (Figure 10A) and keratinocytes (Figure 10B) may be due to the high surfactant (PL) concentration in the formulations.

However, when annatto oil, which has no toxic action, is encapsulated, the cytotoxicity occurs only at higher concentrations, suggesting that the compounds present in AO could have a protective effect on skin cells. In fact, bixin, the main component of AO, is found to present antioxidant and antigenotoxic activities [95,96].

Table 4 presents the results of the half-maximal inhibitory concentration (IC_50_) of each nanoparticle, determined by measuring the cytotoxicity (dose-dependent) displayed in Figure 10 by the percentage of cell viability. The SLNcp samples demonstrated higher values of IC_50_ with no significant difference between the values calculated for both cell types, requiring 243.78 and 345.50 mg/mL to reduce half the concentration of 3T3 and HaCaT cells, respectively. However, nanostructured carriers were more cytotoxic with an IC_50_ lower than SLN samples. In addition, it was observed that only samples with AO at 2% have statistical differences (*p* < 0.05) between the cell lines evaluated, being more toxic to fibroblasts. With regard to the IC_50_, comparing the different types of nanoparticles in contact with the same cell type, significant differences were observed between all samples (SLN vs. the two concentrations of AO in the NLCs and between NLC2 vs. NLC4) when the assay was performed with HaCaT. However, for 3T3 cells, the IC_50_ values were statistically different only between SLNcp and NLCcp4.

In addition, it is important to emphasize that the concentration that demonstrated efficacy against *L. major,* presented in Figure 9, was considerably lower than the concentrations that caused toxicity to the fibroblasts and keratinocyte (at least 24 times lower) cells also found in connective and skin tissue, respectively, which demonstrates a large margin of safety for the formulations.

## 4. Conclusions

In conclusion, only the lipid nanoparticles prepared with CP and containing AO presented a stable and effective system, acting against one of the causative agents of cutaneous leishmaniasis in concentrations that do not display toxicity fibroblast and keratinocyte cells. Free AO was not effective against amastigote forms of *L. major*, present in macrophages. In this way, the AO encapsulation process in lipid-based carriers facilitated the internalization of the drug to eliminate parasites into the cell. Therefore, this system opens good prospects in developing a new drug based on lipid systems containing components extracted from the Annatto to assist in treating cutaneous Leishmaniasis. It can be a promising alternative in the treatment of leishmaniasis, allowing accessibility and convenience.

## Figures and Tables

**Table 1:** Composition of lipid nanoparticles.

Samples ^1^	CP (%)	AO (%)	PL (%)	Water (%)
SLNcp	10	-	11.7	78.3
NLCcp2	10	2	11.7	76.6
NLCcp4	10	4	11.7	74.8
	**MM (%)**	**AO (%)**	**PL (%)**	**Water (%)**
SLNmm	10	-	11.7	78.3
NLCmm2	10	2	11.7	76.6
NLCmm4	10	4	11.7	74.8

Legend: cetyl palmitate, CP; myristyl myristate, MM; synperonic™ PE, PL; annatto oil fraction (AO). ^1^ Sample’s concentration (%, *w*/*w*).

**Table 2:** DSC analysis of lipid nanoparticles produced with cetyl palmitate and myristyl myristate and their compounds (PL, CP, and MM).

Samples	1st Curve			2nd Curve		
	T_Onset_ (°C)	T_Endset_ (°C)	Enthalpy ∆H (mJ/mg)	T_Onset_ (°C)	T_Endset_ (°C)	∆H (mJ/mg)
PL	50.17	58.85	−162.31	-	-	-
CP	50.08	57.30	−184.76	-	-	-
SLNcp	50.88	57.58	−144.45	-	-	-
NLCcp2	49.56	55.73	−127.93	-	-	-
NLCcp4	49.76	55.43	−116.55	-	-	-
MM	34.59	43.08	−172.35	-	-	-
SLNmm	35.14	41.37	−108.42	47.39	54.96	−87.30
NLCmm2	33.09	40.86	−105.73	47.61	55.01	−84.31
NLCmm4	33.59	39.66	−80.78	46.24	53.49	−71.06

**Table 3:** Encapsulation efficiency of NLCs based on Cetyl Palmitate.

Sample	EE%	AO Encapsulated (g)
NLCcp2	78.92 ± 2.89	0.40
NLCcp4	50.54 ± 3.41	0.51

Mean ± sd, *n* = 6.

**Table 4:** IC_50_ of lipid nanoparticles in 3T3 and HaCaT culture cells.

Cells	IC_50_ (mg/mL)		
	SLNcp	NLCcp2	NLCcp4
BALB/c 3T3	243.78 ± 23.11 ^a^*	181.93 ± 8.67 ^e^*	153.64 ± 7.63
HaCaT	345.50 ± 59.51 ^b^*^,c^*	257.11 ± 42.11 ^d^*	123.37 ± 24.98

**Statistics:** ANOVA with a Tukey-Kramer post-test was used in the comparison of groups of formulations related to each cell line; for that, it was considered: BALB/c 3T3: ^a^ SLNcp vs. NLCcp4; HaCaT: ^b^ SLNcp vs. NLCcp2; ^c^ SLNcp vs. NLCcp4; ^d^ NLCcp2 vs. NLCcp4. Test t Student was used for IC_50_ of nanoparticles between the two types of cell line: ^e^ NLCcp2: IC_50_ BALB/c 3T3 vs. IC_50_ HaCaT. Significance (*) was considered *p* < 0.05 for the test. Mean ± sd, *n* = 6.

## Supplementary Materials

The following are available online at https://www.mdpi.com/article/10.3390/pharmaceutics13111912/s1, Figure S1: Calibration curve of AO in ethanol. The absorbance was recorded and the calibration curve plotted against concentrations of AO, which followed Beer’s law and gave a straight line with R^2^= 0.9994 and equation y = 7.2416x − 0.0221; Figure S2: Transmission electron microscopy of samples, SLNmm (a), NLCmm2 (b), and NLCmm4 (c), shows the morphology and size of the nanoparticle lipid based. The images (46,460x magnification) give polydispersity and detail some nanoparticles; Figure S3: Diffractograms of myristyl myristate. Red arrow shows a small peak in 7°; Table S1: Particle size, polydispersity index, and zeta potential of CP and MM nanoparticles in different storage times; Table S2: Main reflections and lattice spacings of the CP, MM and SLN/NLC calculated by Bragg equation. α is considered the most unstable form, d values between 0.415 and 0.42 nm, β stable forms, d = 0.46 nm and β’ with 0.42 < d < 0.43 nm or 0.37 < d < 0.40 nm.
