# Supplementary material for: Annatto Oil Loaded Nanostructured Lipid Carriers: A Potential New Treatment for Cutaneous Leishmaniasis
Source: Pharmaceutics. 2021 Nov 11;13(11):1912. doi: 10.3390/pharmaceutics13111912 (PMC8618414; doi:10.3390/pharmaceutics13111912)
Supplement: Supplementary file 1 [file pharmaceutics-13-01912-s001.zip › pharmaceutics-1358361-supplementary.pdf]

pharmaceutics

MDPI

Supplementary Materials: Annatto Oil Loaded Nanostructured Lipid Carriers: A Potential New Treatment for Cutaneous Leishmaniasis

Marianna Araújo Ferreira , Renato Ferreira de Almeida Júnior , Thiago Souza Onofre , Bruna Renata Casadei , Kleber Juvenal Silva Farias , Patricia Severino , Camilo Flamarion de Oliveira Franco , Fernanda Nervo Raffin , Túlio Flávio Accioly de Lima e Moura and Raquel de Melo Barbosa

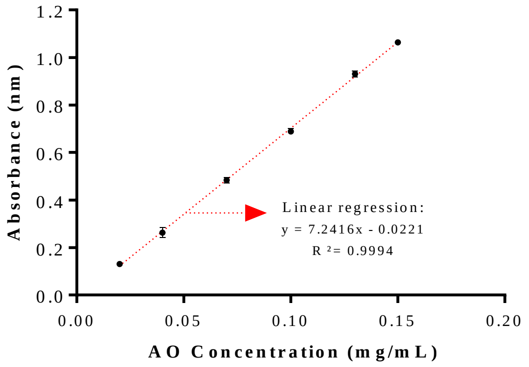

Figure S1. Calibration curve of AO in ethanol. The absorbance was recorded and the calibration curve plotted against concentrations of AO, which followed Beer's law and gave a straight line with R^2= 0.9994 and equation y=7.2416x - 0.0221.

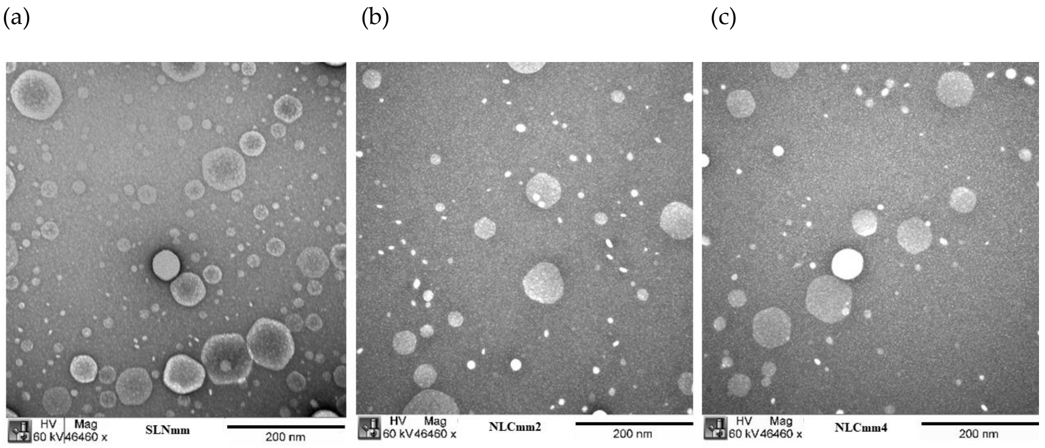

Figure S2. Transmission electron microscopy of samples, SLNmm (a), NLCmm2 (b), and NLCmm4 (c), shows the morphology and size of the nanoparticle lipid based. The images (46,460x magnification) give polydispersity and detail some nanoparticles.

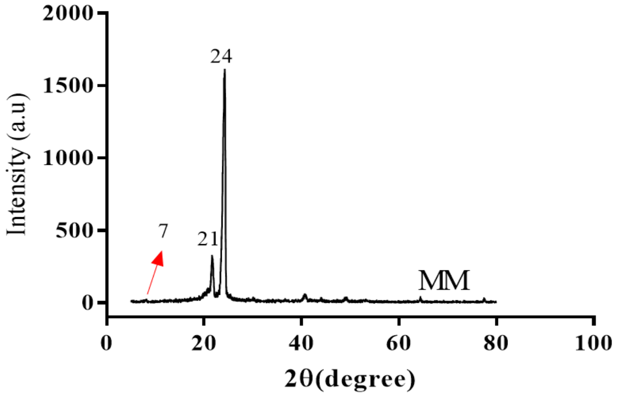

Figure S3. Diffractograms of myristyl myristate. Red arrow shows a small peak in 7°

Table S1. Particle size, polydispersity index, and zeta potential of CP and MM nanoparticles in different storage times.

	Time (days)	SLNcp	NLCcp2	NLCcp4	SLNmm	NLCmm2	NLCmm4
	1	173.7 ± 1.2[a]	184.8 ± 1.0[a]	172.6 ± 1.0	176.6 ± 2.2	189.6 ± 1.4	170.3 ± 2.7
	30	178.0 ± 1.7	189.4 ± 0.7	177.3 ± 1.3	170.5 ± 0.5	180.9 ± 1.1	175.4 ± 0.6
Size	60	174.1 ± 0.2	184.9 ± 1.0	173 5 ± 1.7	173.4 ± 0.3	-	-
(nm)	90	181.0 ± 0.7[a]	190.1 ± 2.4[a]	173.9 ± 1.4	171.2 ± 0.6	-	-
	1	0.12 ± 0.01	0.19 ± 0.01	0.19 ± 0.01	0.21 ± 0.03	0.21 ± 0.03	0.20 ± 0.03
	30	0.14 ± 0.02	0.16 ± 0.00	0.19 ± 0.01	0.23 ± 0.02	0.25 ± 0.01	0.27 ± 0.06
	60	0.10 ± 0.01	0.17 ± 0.02	0.18 ± 0.02	0.22 ± 0.05	-	-
PDI	90	0.11 ± 0.01	0.17 ± 0.02	0.19 ± 0.00	0.23 ± 0.01	-	-
	1	-23.07 ± 0.1[b]	-39.13 ± 0.5[b]	-33.23 ± 0.9	-15.56 ± 0.88	-40.72 ± 1.23	-42.21 ± 0.67
	30	-20.30 ± 0.6	-29.80 ± 0.6	-37.40 ± 0.2	-17.8 ± 0.67	-44.80 ± 0.50	-41.80 ± 0.50
Zeta	60	-14.27 ± 0.4	-34.43 ± 0.5	-37.00 ± 2.9	-17.0 ± 0.70	-	-
(mV)	90	-12.77 ± 0.5[b]	-30.83 ± 0.2[b]	-34.50 ± 0.3	-16.50 ± 1.00	-	-

Statistics: Mean ± SD, $n = 3$. The results of each sample obtained throughout the storage were analyzed through ANOVA, with a Tukey-Kramer post-test. ‘a’ represents a statistical difference of day one *vs.* day ninety of the SLN and NLCcp2 for particle size; ‘b’ day one *vs.* day ninety of SLN and NLCcp2 for zeta potential. Significance was considered p <0.05 for all tests. Observed a phase separation of NLCmm2 and NLCmm4 after 60 days of storage.

Table S2. Main reflections and lattice spacings of the CP, MM and SLN/NLC calculated by Bragg equation. α is considered the most unstable form, d values between 0.415 and 0.42nm, β stable forms, d = 0.46 nm and β′ with 0.42 < d <0.43 nm or 0.37 < d < 0.40 nm.

	Angle 2θ experimental (°)	Lattice spacings d (nm)
MM	20.82	0.43 - β′
	24.26	0.37 - β′
SLNmm	21.72	0.41 - α
	23.44	0.38 - β′
NLCmm2	21.44	0.41 - α
	23.04	0.39 - β′
NLCmm4	21.80	0.41 - α
	23.34	0.38 - β′
CP	21.49	0.41 - α

	23.74	0.37 - β′
SLNcp	21.51	0.41 - α
	23.34	0.38 - β′
NLCcp2	21.51	0.41 - α
	23.01	0.39 - β′
NLCcp4	21.47	0.41 - α
	23.07	0.39 - β′
